# Psychiatric disorders, personality and neuropsychological alterations in Erdheim-Chester disease

**DOI:** 10.1186/s13023-022-02609-x

**Published:** 2023-01-11

**Authors:** Charlotte Soumet-Leman, Jean-Yves Rotge, Pauline Delavaud, Zahir Amoura, Fleur Cohen-Aubart, Julien Haroche

**Affiliations:** 1 ICP (EA7403), équipe VCR, Paris, France; 2 Université de Lorraine, APEMAC, équipe EPSAM, Metz, France; 3 Department of Adult Psychiatry, Pitié-Salpêtrière Hospital, AP-HP, Paris, France; 4 Inserm U 1127, CNRS UMR 7225, Brain and Spine Institute (Institut du Cerveau et de la Moelle Epinière), ICM, Paris, France; 5 Sorbonne Université, Paris, France; 6 Internal Medicine Department 2, Pitié-Salpêtrière Hospital, French National Centre for Histiocytoses, AP-HP, Sorbonne Université, Paris, France

## Abstract

Although neurological manifestations and changes in brain volumes have been described in Erdheim-Chester disease (ECD), it remains unknown whether ECD may be associated with psychiatric symptoms and cognitive dysfunctions. We assessed the presence of psychiatric disorders, changes in temperaments and characters, and neuropsychological performances in 32 ECD patients (mean age = 59) younger than 70, not treated with interferon alpha during the last 6 months, and without other serious illnesses. ECD patients exhibited high level of past depressive disorder (80%) and anxiety disorder, especially agoraphobia (29%). They revealed personality changes, especially with high agreeableness (t = 3.18, *p* < 0.005) and high conscientiousness (t = 3.81, *p* < 0.001). Neuropsychological assessments showed impairments in attention (GZ: t = 16.12, *p* < 0.0001, KL: t = 37.01, *p* < 0.0001) and episodic memory performances (STIR: t = − 3.01, *p* = 0.006, LTFR: t = − 2.87, *p* = 0.008, LTIR: t = − 3.63, *p* = 0.001). Executive functions, such as flexibility, inhibitory control, were unimpaired. Although it remains to be clarified whether these psychiatric symptoms and cognitive impairments may impact the daily functioning and the quality of life, the present study highlights the need to consider cognitive and emotional states in ECD management.

## Key points

- ECD is associated with a great lifetime prevalence of depressive and anxiety disorders.
- ECD is also associated with changes in personality and in neuropsychological performances.

## Introduction

Erdheim-Chester disease (ECD) is a rare histiocytic neoplasm in the L group from the 2016 WHO revised classification of histiocytosis, characterized by xanthomatous or xanthogranulomatous infiltration of tissues by foamy histiocytes. This multi-system disease virtually infiltrates any organs and system and has heterogeneous presentations. The most frequent clinical manifestations include bone pain, peri-aortic infiltration, pericardial infiltration, exophthalmos, diabetes insipidus, pulmonary infiltration or involvement of the central nervous system [10, 11]. ECD is also associated with cytokine elevations and perturbations, which may be responsible for inflammatory symptoms, such as fever, sweats or fatigue [1].

Diamond et al. [9] have listed symptoms reported by patients and highlighted the high prevalence of psychiatric and neuropsychological symptoms. Among 50 ECD patients, 92% self-reported neuro-psychologic symptoms, especially depression, sadness, anxiety, fatigue or memory problems, all of them impacting greatly their quality of life. These symptoms remain poorly described and there is still no report of the prevalence of psychiatric disorders according to standardized diagnostic criteria or measurement of neuropsychological performances. Here we assessed (1) the presence of psychiatric disorders, according to DSM-IV criteria, (2) the changes in temperaments and characters, and (3) the neuropsychological performances in 32 ECD patients.

## Methods

### Participants

32 patients with a confirmed diagnosis of ECD were recruited in the French National Reference Center of Histiocytoses in Pitié-Salpêtrière Hospital, Paris, France. At the moment of the study, this Reference Center followed a population of 165 ECD patients described in Cohen-Aubart et al. [6].Patients had to be 70 or less (1), not to be treated with interferon alpha during the last 6 months (2), not to suffer from another general disease, such as cancer (3), not to suffer from current depressive episode (4) and to provide an informed and written consent (5).

### Psychiatric assessments

The presence of past or current psychiatric disorders was assessed with the French version of the Mini International Neuropsychiatric Interview [16] on the basis of DSM-IV [8] criteria.

### Personality assessments

ECD patients had to fulfill the 45-items French version of the Big Five Inventory (BFI) scale, which assess five broad personality dimensions, namely extraversion, agreeableness, conscientiousness, openness and neuroticism [14].

### Neuropsychological assessments

Neuropsychological performances were evaluated with the French validated versions of the following tests:

1. the WCST assessed executive functions, such as planning, shifting and cognitive flexibility [12]. Four stimulus cards with symbols differing in color, shape and number were presented. Participants were required to match a fifth card to one of the 4 stimulus cards on the basis of color, shape or number and to keep or change their strategy. The total number of errors, non-perseveration and perseveration errors were recorded.
2. the D2 Test measured selective and sustained attention [2]. Participants were asked to cross out target letters "d" with two marks among distractors ("p" or "q" with two marks or "d" with 1 or 3 marks). The time was 20 s by line. The performances indices were: (i) KL, the number of targets correctly canceled, (ii) GZ, the quantitative performance index, i.e. the total number of characters treated, and (iii) GZ-F, the corrected quantitative performance.
3. the Stroop Test assessed focused attention and inhibitory control, requiring word reading, color naming and interference conditions (non-matching words and colors) [15].
4. the CVLT [17] assessed verbal learning, verbal memory and verbal organizational strategies. The test was based on a list of 16 items with an imbedded semantic structure and an interference list B. Measures consisted in immediate free/cued recall trial on list A (STFR/STIR) and long-delay free/clued recall trials on list A (LTFR/LTIR) 20–30 min later.
5. the verbal fluency tests were performed to assess semantic and phonetic fluency in 2 min, which may be considered as an index of executive commitment.
6. the Digit Span test was used to assess verbal short-term working memory. After having read digits, participants were asked to repeat them in three orders: forward, backward and sequencing.
7. the TMT-A and -B assessed mental flexibility and executive functions. TMT-A required to draw lines sequentially connecting 25 encircled numbers distributed on a sheet of paper. In TMT-B, participants were asked to alternate between numbers and letters (e.g., 1, A, 2, B, 3, C, etc.).

### Data analysis

One-sample t-tests was conducted to compare the mean values with a reference value. For personality tests, mean BFI scores for the French general population was used as reference values [14]. For neuropsychological characteristics, individual Z-scores were compared with French population norms for gender, age and education level. We limited the risk of false-positive results by applying Bonferroni correction.

## Results and discussion

The 32 patients included in the present study had very similar clinical characteristics of the whole ECD population (n = 165) followed in the Reference Center [6]. They were randomly proposed to participate to the present study without any consideration else than inclusion criteria. There was some slight, but not clinically significant, differences between both populations regarding gender (women: 47% in the included sample vs 28% in the whole population), age at first symptoms (50 vs 52), BRAF V600E status (62% vs 53%), and regarding cerebral MRI patterns (tumoral patterns: 59% vs 66%, pseudo-degenerative patterns: 47% vs 50%, vascular patterns: 22% vs 18%).

Of the 32 ECD patients (mean age: 58.7, SD: 9.3, 15 women), 3 were taking antidepressant and 10 benzodiazepine at the moment of the study. Main clinical characteristics were reported in Table 1 and psychiatric comorbidities were given in Table 2. ECD patients exhibited high level of past depressive disorder (80%) and anxiety disorder, especially agoraphobia (29%). The level of depression was not explained by depressogenic effects of interferon alpha, since among the 8 patients without history of interferon alpha 7 had a past history of depression (87.5%).

**Table 1:** Main clinical characteristics of the ECD population (n = 32)

	n (%)
BRAF V600E mutation	20 (62.5)
Age at first symptoms (mean[SD])	49.6 [14.2]
*Current treatment*	
Steroid	6 (18.8)
Interferon alpha	2 (6.3)
Vemurafenib	12 (37.5)
Cobimetinib	4 (12.5)
Others	5 (15.6)
*Past treatment*	
Steroid	13 (40.6)
Interferon alpha	24 (75)
Vemurafenib	12 (37.5)
Cobimetinib	7 (21.9)
Others	13 (40.6)
Cerebral MRI patterns*	
MRI considered as normal	8 (25)
Tumoral patterns	19 (59.4)
Pseudo-degenerative patterns	15 (46.9)
Vascular patterns	7 (21.9)

*ECD* Erdheim-Chester disease

*MRI patterns was described on the basis of prior publications (see reference Cohen-Aubart et al. [7])

**Table 2:** Frequency of psychiatric disorders in ECD (n = 32)

	n (%)
Past major depressive episode	25 (80)
Current major depressive episode	0 (0)
Past hypomanic/manic episode	0 (0)
Current hypomanic/manic episode	0 (0)
Panic disorder	1 (3)
Agoraphobia	9 (29)
Social anxiety disorder	0 (0)
Obsessive–compulsive disorder	0 (0)
Posttraumatic stress disorder	0 (0)
Alcohol dependence	0 (0)
Alcohol abuse	0 (0)
Substance dependence	1 (3)
Substance abuse	0 (0)
Psychotic disorder	0 (0)
Anorexia nervosa	1 (3)
Bulimia nervosa	0 (0)
Generalized anxiety disorder	3 (10)
Antisocial personality disorder	0 (0)

*ECD* Erdheim-Chester disease

Personality evaluations revealed personality changes, especially with high agreeableness and high conscientiousness, as compared with general population (Table 3).

**Table 3:** Personality evaluation and neuropsychological test performances in ECD patients

	Mean	SD	Reference	*t*	*p*
*Personality evaluation* (BFI, n = 30)					
Extraversion	3.10	0.94	3.2	− 0.58	0.56
Agreeableness	4.19	0.49	3.9	3.18	< 0.005*
Conscientiousness	3.77	0.53	3.4	3.81	< 0.001*
Openness	3.40	0.69	3.5	1.61	0.12
Neuroticism	2.72	0.95	3.0	− 0.79	0.44
*Neuropsychological test performance* (n = 25)					
Wisconsin card sorting task					
Total number of errors	0.06	1.81	0	0.16	0.88
Non-perseveration errors	− 0.75	1.86	0	− 2.04	0.05
Perseveration errors	− 0.29	2.80	0	− 0.53	0.60
d2 test of attention					
KL	96.15	13.25	100	37.01	< 0.0001*
GZ	84.40	20.02	100	16.12	< 0.0001*
GZ-F	86.40	20.79	100	15.99	< 0.0001*
Stroop Test					
Word reading	− 0.83	1.48	0	− 2.87	0.024
Color naming	− 0.81	1.72	0	− 2.87	0.008*
Stroop interference	− 0.24	1.31	0	0.92	0.37
California verbal learning test					
STFR	− 0.63	1.33	0	− 2.38	0.025
STIR	− 0.84	1.40	0	− 3.01	0.006*
LTFR	− 0.83	1.44	0	− 2.87	0.008*
LTIR	− 1.01	1.39	0	− 3.63	< 0.001*
Verbal fluency tests					
Semantic	− 0.48	1.08	0	− 2.29	0.03
Phonetic	− 0.24	1.14	0	− 1.07	0.29
Digit span test					
Forward	− 0.47	0.71	0	− 3.36	0.0026*
Backward	− 0.23	0.84	0	− 1.37	0.18
Sequencing	− 0.22	0.86	0	− 1.33	0.20
Trail making test					
TMT-A	− 0.63	1.74	0	− 1.86	0.07
TMT-B	− 1.54	4.34	0	− 1.80	0.08

*ECD* Erdheim-Chester disease, *SD* standard deviation, *BFI* Big Five Inventory

*Significant after Bonferroni correction

**Fig. 1:**
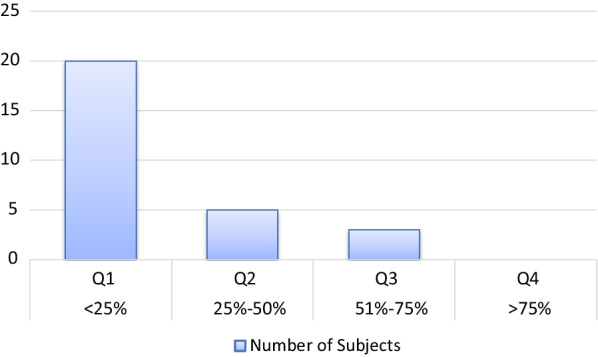
Distribution of D2 scores in ECD (n = 25)

**Fig. 2:**
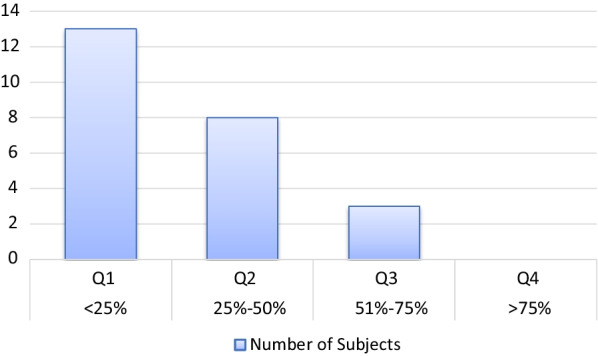
Distribution of CVLT scores in ECD (n = 24)

Neuropsychological assessments showed impairments in attention and verbal memory (Table 3): on the D2 test, 80% of patients obtained scores in the lowest 25% compared to patients of the same age (Fig. 1). On the CVLT, they were 54% in this case (Fig. 2), indicating the importance of the deficit in these abilities. In order to assess whether neuropsychological results might be partially explained by depressive symptoms, we tested correlations between neuropsychological variables and BDI scores and no significant relationship was found (all *p*-values > 0.05).

These results indicated high levels of depressive and anxiety disorders, but also some subtle cognitive and emotional impairments with some personality changes and neuropsychological deficits in sustained attention and episodic memory. Executive functions, such as flexibility, inhibitory control, were unimpaired. A study on a larger number of subjects would be necessary to characterize these disorders more precisely, but they seem to differ in nature from the cognitive disorders observed in other severe diseases such as cancer [13] or multiple sclerosis [3].

ECD was associated with a great lifetime prevalence of depressive and anxiety disorders. This association was not explained by the depressogenic effects of treatments. Regarding the relationship between mood and inflammation, it could be assumed that the high level of depression would be related to inflammatory processes in ECD. Inflammatory states induced changes in mood, by affecting tryptophan's metabolism [5]. Tryptophan's metabolites are known to respectively contribute to neuronal excitotoxicity or protection, which are dysregulated in depression [5]. To explore the depressogenic mechanisms in ECD, the tryptophan pathways could be specifically assessed.

ECD was also associated with changes in personality and in neuropsychological performances. It remains unclear whether these impairments are related to the history of depressive disorders or whether they are more specifically related to ECD. Although no relationship was found with current depression scores, these cognitive and emotional changes could be viewed as residual symptoms of past depressive episodes. Such cognitive deficits were described to be responsible for great psychosocial dysfunctions in daily life with a great impact in quality of life [4].

The present study showed the importance of neuropsychological and psychiatric symptoms in ECD, but also open new therapeutic perspectives to improve daily functioning among ECD patients. We propose to investigate the benefit of the cognitive remediation in ECD. Further studies should explore pathophysiological pathways involved in the association between ECD and depression, but also explore the clinical consequences and the therapeutic strategies of psychiatric symptoms.

## Data Availability

The datasets analyzed during the current study are available from the corresponding author on reasonable request.

## Abbreviations

BFI: Big Five Inventory
CVLT: California verbal learning test
DSM-IV: Diagnostic and statistical manual of mental disorders-4th edition
ECD: Erdheim-Chester disease
GZ: Global score (D2 test)
GZ-F: Global score-faults
KL: Concentration level
LTIR: Long term indexed recall
LTFR: Long term free recall
MRI: Magnetic resonance imaging
STIR: Short term indexed recall
STFR: Short term free recall
TMT: Trail making test
WCST: Wisconsin card scoring test
